# Application of data-driven blended online-offline teaching in medicinal chemistry for pharmacy students: a randomized comparison

**DOI:** 10.1186/s12909-024-05701-x

**Published:** 2024-07-09

**Authors:** Yong-ming Zhao, Si-si Liu, Jin Wang

**Affiliations:** 1 Department of Pharmacy, Hebei North University, Zhangjiakou, China; 2 Hebei Key Laboratory of Neuropharmacology, Zhangjiakou, China

**Keywords:** Data-driven, Online-offline teaching, Blended teaching, Lecture-based learning, PBL, Medicinal chemistry

## Abstract

**Background:**

The purpose of this study was to evaluate the effectiveness and efficiency of implementing a data-driven blended online-offline (DDBOO) teaching approach in the medicinal chemistry course.

**Methods:**

A total of 118 third-year students majoring in pharmacy were enrolled from September 2021 to January 2022. The participants were randomly assigned to either the DDBOO teaching group or the traditional lecture-based learning (LBL) group for medicinal chemistry. Pre- and post-class quizzes were administered, along with an anonymous questionnaire distributed to both groups to assess students’ perceptions and experiences.

**Results:**

There was no significant difference in the pre-class quiz scores between the DDBOO and LBL groups (*T*=-0.637, *P* = 0.822). However, after class, the mean quiz score of the DDBOO group was significantly higher than that of the LBL group (*T* = 3.742, *P* < 0.001). Furthermore, the scores for learning interest, learning motivation, self-learning skill, mastery of basic knowledge, teamwork skills, problem-solving ability, innovation ability, and satisfaction, as measured by the questionnaire, were significantly higher in the DDBOO group than in the traditional group (all *P* < 0.05).

**Conclusion:**

The DDBOO teaching method effectively enhances students’ academic performance and satisfaction. Further research and promotion of this approach are warranted.

**Supplementary Information:**

The online version contains supplementary material available at 10.1186/s12909-024-05701-x.

## Introduction

With the comprehensive integration of information technology in the field of education, traditional classrooms have evolved with various new models of online teaching, making the instructional process more dynamic and effective [1]. Learners are encouraged to engage in online learning tasks and digital game-based activities, experiencing the joy of dealing with digital challenges, acquiring knowledge, and enhancing learning outcomes [2, 3]. These emerging technologies serve as crucial tools for information dissemination in online education, profoundly impacting the reform of medical school education [4].

Blended learning, an instructional model combining digital online learning with face-to-face classroom teaching, has gradually drawn more attention with advancements in internet technology and education [5, 6]. The concept of blended learning was first introduced in the U.S. National Education Technology Plan. Since 2004, the United States has been actively adopted and innovated the blended learning approach, continually exploring advancements in technology and other aspects.

Higher education has undergone a significant evolution in teaching paradigms. Following eras of experiential imitation teaching and computer-assisted instruction [7], the current landscape is increasingly characterized by the data-driven instruction [8]. This approach incorporates next-generation information technologies such as the Internet of Things, big data, cloud computing, and mobile internet, involving systematic collection and analysis of both online and offline learning data to inform instructional improvements and elevate learning outcomes [9].

Data-driven instruction is an innovative approach that harnesses diverse forms of data to shape and enhance teaching practices [10]. This encompasses a spectrum from summative data, such as test scores, to formative data gauging student understanding through activities like discussions. Diverging from summative assessments primarily designed for assigning grades, formative assessments aim to refine teaching methods. The collection and analysis of both types of data empower educators to discern patterns and address shortcomings within their classrooms. Through the strategic utilization of these insights, educators can tailor instruction to individual student needs, pinpoint specific areas for improvement, and implement timely interventions to bolster overall student success. This proactive and personalized approach to teaching ensures that educators are equipped with the necessary information to optimize learning experiences and foster positive educational outcomes for every student.

Medicinal chemistry is a comprehensive discipline focused on the discovery and invention of new drugs, the synthesis of chemical pharmaceuticals, elucidating the chemical properties of drugs, and researching the interaction patterns between drug molecules and cellular entities. Its scope encompasses the chemical structure, physicochemical properties, preparation methods, transport metabolism, structure-activity relationships, chemical mechanisms of drug action, as well as approaches and methods for the discovery of new drugs [11]. With the continuous deepening of educational reforms, the teaching approach in medicinal chemistry has shifted from traditional methods towards a blended learning model [12, 13]. This approach seamlessly integrates online and offline teaching, leveraging the advantages of interactive communication in face-to-face classrooms while overcoming the limitations of traditional offline teaching, such as a singular format and limited content. Moreover, the use of online resources in the blended learning model has expanded the platform for medicinal chemistry education, greatly enriching the teaching content. It has not only sparked students’ interest in learning but also broadened their perspectives. Therefore, it is essential to explore the application of the blended learning model in biochemistry teaching.

Teaching medicinal chemistry presents a unique challenge for pharmacy students, prompting a preliminary investigation into the data-driven blended online-offline teaching model’s implementation. This instructional approach amalgamates various teaching techniques with the objective of improving students’ learning outcomes and satisfaction, thereby offering additional teaching avenues for nurturing pharmaceutical talent.

## Methods

### Participants

This teaching reform experiment is open to all third-year Pharmacy students at Hebei North University. Before commencing the experiment, students were required to complete a short screening questionnaire to ensure they had the necessary resource for the experiments. The questionnaire asked the following five yes-or-no questions: (1) Do you have a stable internet connection? (2) Do you have access to an independent electronic device (laptop, tablet, or smartphone)? (3) Are you able to complete the online course? (4) Are you able to complete the exams and questionnaires? (5) Are you aware of this experiment and willing to participate? Students who answered “yes” to all questions were eligible for the study, while those who answered “no” to one or more questions were excluded.

### Sample size, grouping and blinding methods

According to the sample size calculation method reported in the literature [14], the study required a minimum of 52 participants per group to achieve a significance level (α) of less than 0.05 and a power (1-β) of 80%. The participants were randomly divided into experimental group (*n* = 59) and control group (*n* = 59) using a simple randomization. Both groups were supervised by the same teaching team, including one professor and two assistants. The experiment was conducted using a single-blind method and the students were blinded after assignment to interventions.

### Study design

We have employed a randomized controlled trail to assess the effectiveness of a data-driven blended online-offline (DDBOO) teaching model on a group of healthy volunteers. The DDBOO method was implemented in the experimental group, while the control group received the traditional lecture-based learning (LBL).

### Interventions

#### The DDBOO model for medicinal chemistry course

**Fig. 1:**
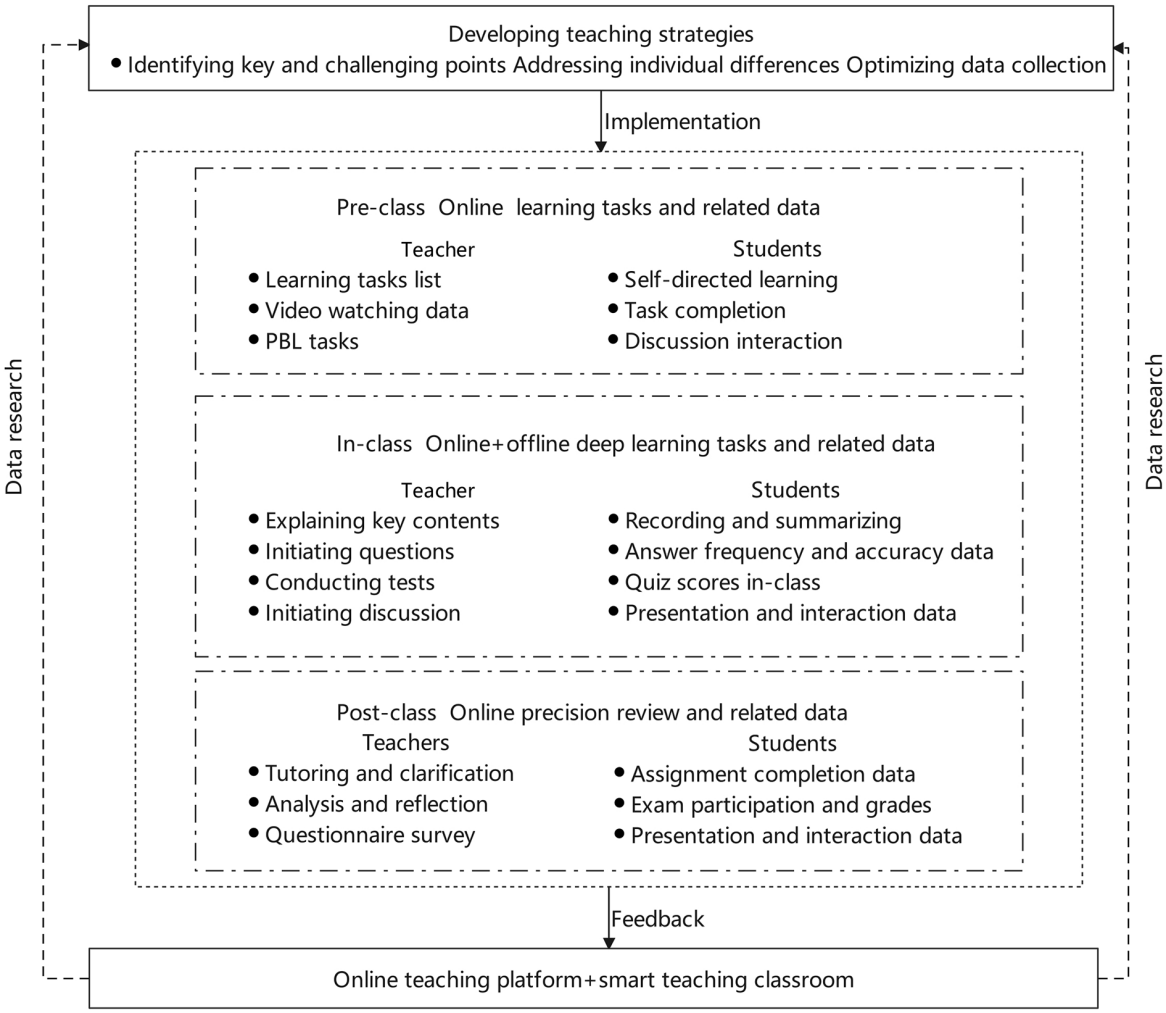
An overview of the study design

The DDBOO instructional process is structured into three phases: pre-class, in-class, and post-class. Through a seamless integration of synchronous and asynchronous learning, we have formulated a comprehensive DDBOO teaching approach, as illustrated in Fig. 1.

##### Before class

The teacher introduces the theme, characteristics and tasks of the lesson online, emphasizing the importance of the chapter and sparking student’s interest. Students engage in self-directed online learning tasks utilizing the SuperStarLearn software. They access and complete tasks at their own pace, view microlecture videos covering key topics, and subsequently undergo corresponding chapter tests. Following this, Problem-based learning (PBL) scenarios are introduced, encouraging collaborative teamwork to address PBL tasks. For those who do not complete assigned tasks, the learning alert system prompts them to do so. Teachers analyze online learning data, including the duration and frequency of student video views and chapter test accuracy, to identify common issues and pinpoint teaching challenges.

##### In class

During the class, teachers provide comprehensive explanations for commonly challenging issues and assess the learning outcomes through features such as quick response and in-class quizzes on the SuperStarLearn platform. Group discussions and collaborative thinking are encouraged to achieve a deeper understanding. Teachers also provide individualized guidance to address specific issues encountered by students during the learning process. By analyzing learning behaviors, such as participation in quick response and thematic discussions, as well as statistical data from in-class quizzes and assessments of group tasks, teachers can determine student engagement, personalized challenges, and learning effectiveness. This analysis enables teachers to intervene promptly, making adjustments to the teaching pace as necessary.

##### Post class

At the end of the class, the students completed a post-quiz and a questionnaire consisting of nine questions. Following the class, learning data retrieved from the SuperStarLearn Platform reports are used to distribute personalized assignments. By analyzing data such as assignment accuracy, teachers identified cognitive gaps and deviations among students. This information allows for targeted supplementation and correction in the subsequent class.

#### LBL method for medicinal chemistry course

In the control group, the same topics were presented through LBL. The lectures comprised two sessions, conducted once a week for 90 min each. During the class, the routine included the teacher explaining the learning objectives (5 min), delivering the content using PowerPoint slides (65 min), engaging in exercises (10 min), and participating in a class discussion or question-and-answer session (10 min). Students had the opportunity to participate in a question-and-answer session during the lecture, and discussions were encouraged if students wished to share their opinions or respond to their peers’ questions.

#### Outcome measurements

After obtaining informed consent, basic information about the participants, including age and gender, was collected. To evaluate students’ comprehension and application of knowledge, both groups underwent the same assessments, consisting of one pre-quiz and one post-quiz, each lasting 60 min and scored out of 100 points. Additionally, a questionnaire survey was administered at the end of the course to measure students’ self-perceived competence. The details of the questionnaire are presented in the Supplementary materials. This survey covered various aspects such as learning interest, targeted learning, motivation, self-learning skills, mastery of basic knowledge, teamwork abilities, problem-solving proficiency, and innovation capacity. Responses were rated using a 5-level Likert scale: 5 points for “strongly agreed,” 4 points for “agreed,” 3 points for “neutral,” 2 points for “disagreed,” and 1 point for " strongly disagreed.” Furthermore, a survey on satisfaction with the teaching mode was conducted, with responses categorized into four levels: “Very Satisfied,” “Satisfied,” “Neutral,” and “Dissatisfied.” In order to maintain impartial responses, both quizzes and questionnaires were conducted anonymously, mitigating any potential influence, whether positive or negative, on the students.

### Statistical analysis

A chi-squared test (symbolically represented as χ^2^) was employed to assess the discrepancy of count data. To compare two independent groups, the student t-test was utilized. Data were expressed as individual values and as mean ± standard deviation (SD). Statistical analysis was conducted using IBM SPSS statistics 20.0 software. The significance level (alpha) was set to 0.05, and p-values less than 0.05 were considered statistically significant.

## Results

### Baseline characteristics of the students

From September 2020 to January 2021, a total of 118 students actively participated in the teaching experiment. Among them, 46 students were male (38.98%), and 72 students were female (61.02%). The average age of the participants was 20.5 ± 0.7 years. These students were randomly assigned to two groups: the DDBOO group (*n* = 59) and the traditional LBL group (*n* = 59). Notably, all students successfully completed the entire teaching process, including quizzes and questionnaires, and there were no dropouts during the study period. A comprehensive analysis of demographic data between the DDBOO group and the LBL group is presented in Table 1. The results revealed no significant differences between the two groups in terms of gender (*P* = 0.45), age (*P* = 0.673), and pre-quiz scores related to basic knowledge (*P* = 0.822).

**Table 1:** Comparison of basic data between two groups

	DDBOO group	LBL group	t (χ^2^) value	*P* value
Gender				
Male (n,%)	25(42.38%)	21(35.59%)	0.570	0.45
Female (n,%)	34(57.62%)	38(64.41%)		
Age (years)	20.6 ± 0.68	20.5 ± 0.66	0.417	0.673
Pre-quiz about basic knowledge score (point)	78.7 ± 8.8	79.7 ± 9.27	-0.637	0.822

### Comparison of the post-quiz test scores between two groups

**Fig. 2:**
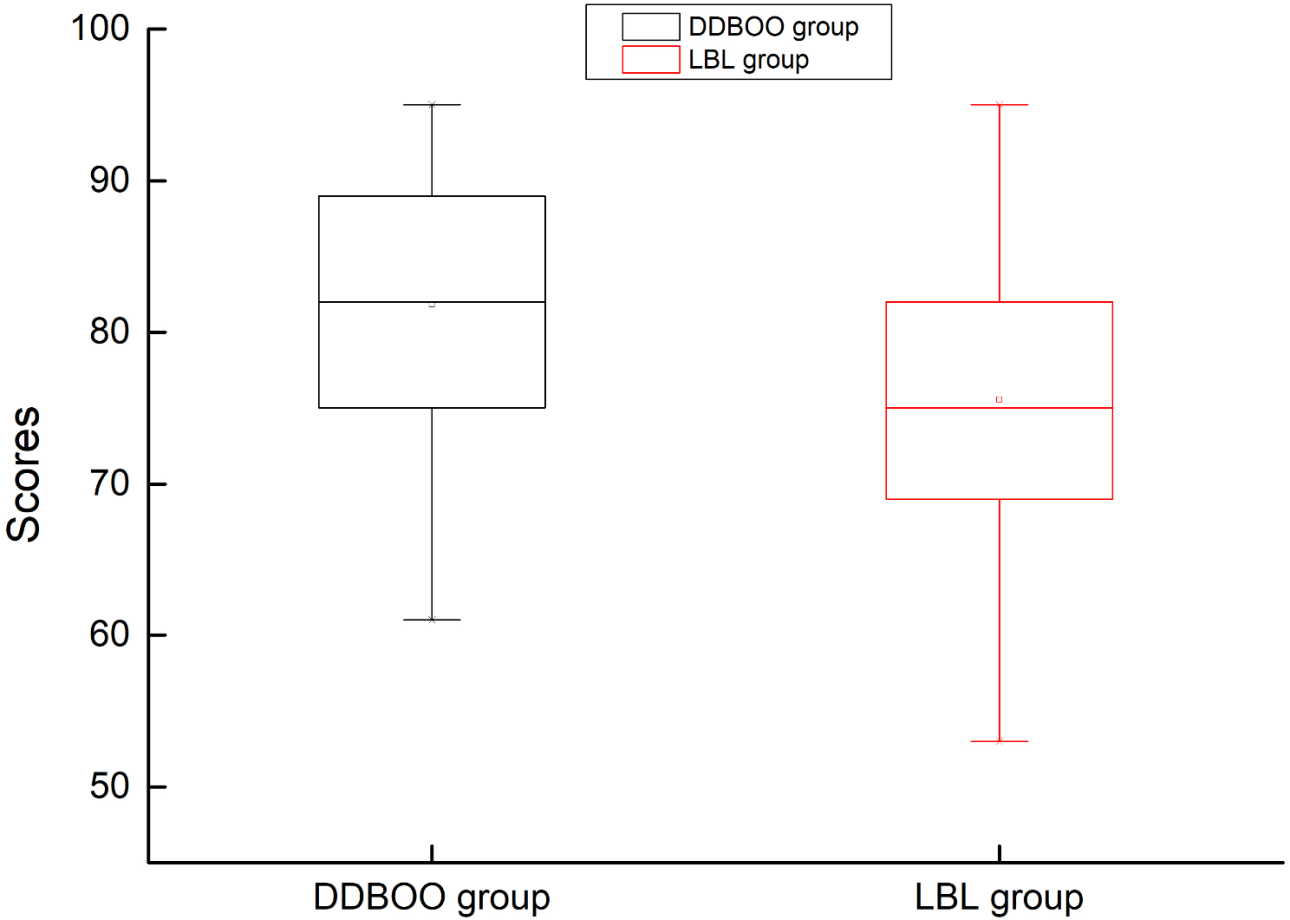
Comparison of the post-class test scores between two groups

As illustrated in Fig. 2, the statistical analysis of the box plots depicting final exam scores reveals that the average scores of the DDBOO group are higher than those of the LBL group(*T* = 3.742, *P* < 0.001). Moreover, there is a reduction in the number of low-scoring students, suggesting a better mastery of professional knowledge among students in the DDBOO group.

### Comparison of self-perceived competence and satisfaction between the two groups

A comprehensive evaluation of the teaching effectiveness between the DDBOO teaching method and the traditional LBL teaching approach was conducted through a post-teaching questionnaire survey. All questionnaires distributed for the survey were successfully collected and proved to be valid. According to Table 2, the DDBOO group outperformed the LBL group in various aspects, including learning interest, learning motivation, self-learning skill, mastery of basic knowledge, teamwork skills, problem-solving ability, and innovation ability, demonstrating statistically significant differences (*P* < 0.05). While the score for learning targeted was higher in the DDBOO group compared to the LBL group, this difference was not statistically significant (*P* > 0.05). Furthermore, as indicated in Table 3, the level of satisfaction within the DDBOO group surpassed that of the traditional LBL group (*P* = 0.011).

**Table 2:** Outcome of questionnaire (agree %)

Statement in questionnaires	DDBOO group (*n* = 58)	LBL group (*n* = 58)	T value	*P* value
Learning interest (point)	4.5 ± 0.6	3.8 ± 0.9	4.673	0.042
Learning targeted (point)	4.3 ± 0.7	3.6 ± 0.9	4.947	0.088
Learning motivation (point)	4.2 ± 0.7	3.5 ± 1.1	4.281	0.000
Self-learning skill (point)	4.1 ± 0.7	3.5 ± 1.2	2.883	0.000
Mastery of basic knowledge (point)	4.3 ± 0.8	3.6 ± 1.0	4.204	0.019
Teamwork skill (point)	4.1 ± 0.8	3.7 ± 1.0	2.646	0.048
Problem-solving ability (point)	4.1 ± 0.9	3.9 ± 1.0	2.655	0.047
Innovation ability (point)	4.0 ± 0.8	3.6 ± 1.2	2.105	0.004

**Table 3:** Comparison of satisfaction with the course between two groups

	Degree of satisfaction			
	Very satisfied (n,%)	Satisfied (n,%)	Neutral (n,%)	Dissatisfied (n,%)
DDBOO group	27(46.55%)	17(29.31%)	10(17.24%)	4(6.90)
LBL group	11(18.97%)	20(34.48%)	19(32.76%)	8(13.79%)
χ^2^ value	11.107			
*P* value	0.011			

**Fig. 3:**
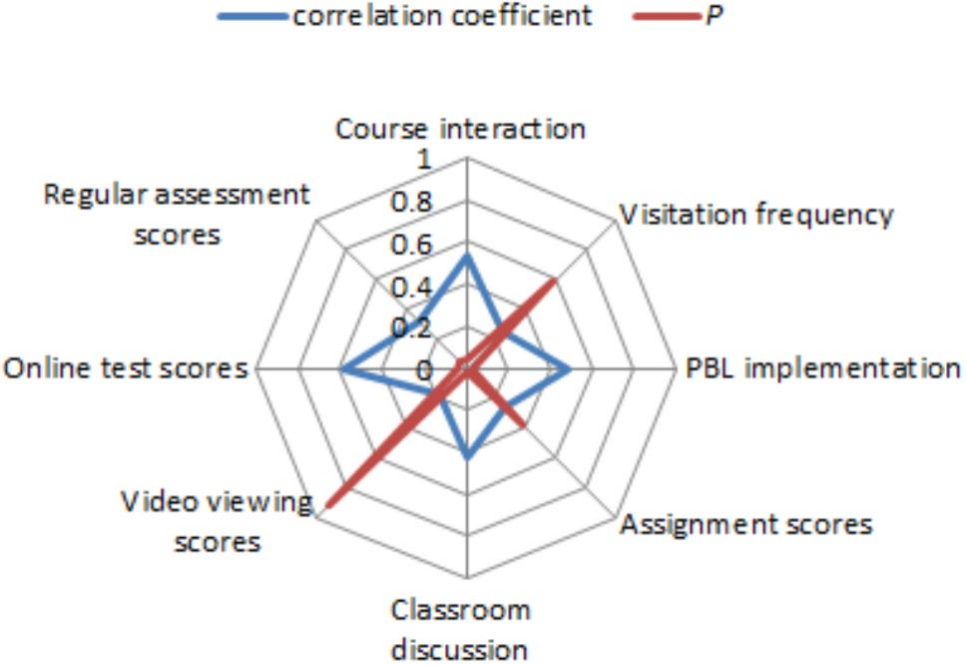
Correlation analysis of learning behaviors

Utilizing SPSS software for data analysis, we conducted a correlation analysis to further examine the relationship between students’ online and offline learning behaviors and their final exam scores under the DDBOO teaching model. The correlation analysis results are depicted in Fig. 3. From Fig. 3, it is evident that the online test scores demonstrates a positively correlation with final exam scores (*r* = 0.52), signifying a noteworthy impact of students’ performance in self-directed learning on overall learning quality. However, the correlations between visitation frequency, online video viewing duration, assignment scores and final scores are not significant. This may be attributed to some students engaging in online activities solely for the purpose of improving their scores. In addition to carefully designing online teaching activities, teachers need to appropriately assign weights to evaluation criteria for online self-directed learning, guiding students towards effective independent learning practices. Regarding offline learning behaviors, course interaction, PBL implementation, and classroom discussions exhibit higher correlations with final exam scores, with correlation coefficients of 0.53, 0.48 and 0.43, respectively. This suggests that classroom interactions and presentation discussions contribute to deepening students’ understanding and mastery of the learned content.

**Fig. 4:**
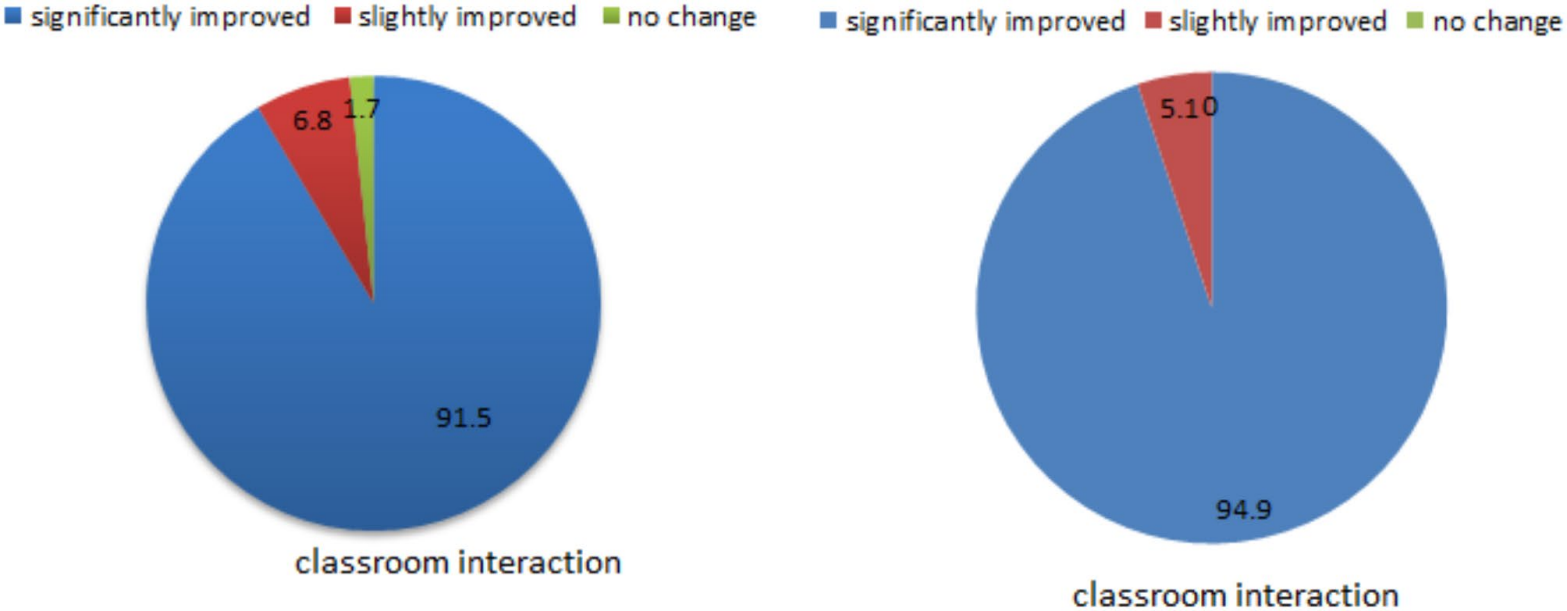
Results of the feedback of DDBOO group

The feedback results from the instructional survey indicate that the DDBOO teaching approach has achieved learning outcomes, as depicted in Fig. 4. 91.5% of students believe that data-driven blended teaching has enhanced their study habit, transformed cognitive patterns, and bolstered subjective initiative. Additionally, 94.9% of students express that classroom interaction is more dynamic, encouraging them to confidently pose questions and articulate their viewpoints. The majority of students acknowledge the significant impact of data-driven blended teaching on overall skill enhancement, particularly in terms of autonomous learning, problem analysis, and teamwork abilities.

## Discussion

This study investigated the effectiveness of a DDBOO teaching approach in medicinal chemistry for pharmacy students. The DDBOO model, integrating online resources with traditional classroom instruction, yielded significant improvements in students’ comprehension application of complex pharmaceutical concepts, and self-perceived competence as measured by post-course surveys. These findings not only highlight the effectiveness of DDBOO model, but also align with existing research on blended learning’s benefits, including flexibility, diverse resources, and enhanced student engagement [15]. Furthermore, DDBOO facilitates real-time feedback, adaptability, and a shift from teacher-centered learning to active problem-solving and collaboration. Data-driven assessments further empower instructors by allowing for early intervention and ongoing refinement of teaching methods based on student performance data [16]. This combined approach paves the way for optimizing student learning and engagement in medicinal chemistry education.

Blended online-offline teaching addresses pharmacy students’ need for practical skills by freeing up classroom time for hands-on practice [17, 18]. Online platforms enable flexible, self-paced learning of theoretical knowledge outside of class, maximizing learning efficiency. This organic integration of theory and practice fosters the development of comprehensive abilities, including operational skills, critical thinking, and innovation. Students appreciate the flexibility and diverse resources offered by the blended approach, leading to increased engagement and enjoyment of the learning process.

The teacher plays a pivotal role in blended online-offline teaching for medicinal chemistry. They design a curriculum integrating both online and offline components, selecting materials tailored to medicinal chemistry education. Utilizing online platforms and resources, teachers engage students in various activities such as discussions and virtual experiments, guiding them through the online learning environment. Moreover, teachers adopt a data-driven approach, collecting and analyzing student performance data to provide individualized support and targeted interventions. Continuous feedback on student performance informs the adaptation of teaching strategies to meet diverse learning needs. Offline sessions, including laboratory work and group discussions, complement online components to offer a comprehensive learning experience. Through these efforts, teachers create a supportive and collaborative environment, fostering student interaction and critical thinking [19]. Overall, the teacher acts as a facilitator, guide, and analyst, utilizing data-driven insights to optimize the blended online-offline teaching approach in medicinal chemistry.

Limitations.

Despite its advantages, the DDBOO teaching model also presents several limitations. One notable limitation is the potential for unequal access to technology and online resources among students, which may widen existing educational inequalities. Additionally, the success of the DDBOO model relies heavily on effective technology integration and teacher training, which may pose challenges for institutions with limited resources or infrastructure. Moreover, the model’s effectiveness may vary depending on factors such as student motivation, prior knowledge, and learning preferences, highlighting the need for further research to better understand its impact across different contexts and populations. Overall, while the DDBOO teaching model offers numerous benefits for enhancing student learning and engagement, careful consideration of its limitations is essential for its successful implementation and long-term sustainability.

## Conclusion

In conclusion, the application of the data-driven blended online-offline teaching model in medicinal chemistry for pharmacy students has demonstrated promising results in enhancing learning outcomes and satisfaction levels. This innovative approach, guided by big data technology, provides a tailored and personalized learning experience that addressed individual student needs. The findings of this study underscore the potential of integrating advanced teaching methodologies with traditional classroom instruction to optimize the educational experience in pharmacy education. Future research should explore the applicability of this blended teaching model to other disciplines, such as clinical medicine, nursing, and public health.

### Electronic supplementary material

Below is the link to the electronic supplementary material.

Supplementary Material 1

## Data Availability

The data used and analyzed during the current study are available from the corresponding author on reasonable request.

## Abbreviations

DDBOO data: Driven blended online-offline
LBL lecture: Based learning

## References

[1] Andrade MS, Miller RM, Kunz MB, Ratliff JM (2022). Online learning in schools of business: what influences faculty to teach online?. Open Learn.

[2] Alzahrani M (2022). Traditional learning compared to Online Learning during the COVID-19 pandemic: lessons learned from Faculty’s perspectives. SAGE Open.

[3] Ahern K (2017). Teaching biochemistry online at Oregon State University. Biochem Mol Biol Educ.

[4] Ramírez-Hurtado JM, Hernández-Díaz AG (2021). Measuring Online Teaching Service Quality in Higher Education in the COVID-19 Environment. Int J Environ Res Public Health.

[5] Asadi N, Khodabandeh F, Yekta RR (2019). Comparing and contrasting the interactional performance of teachers and students in traditional and virtual classrooms of advanced writing course in distance education university. Turk Online J Distan.

[6] Coyle KK, Chambers BD, Anderson PM, Firpo-Triplett R, Waterman EA (2019). Blended learning for sexual Health Education: evidence base, Promising practices, and potential challenges. J Sch Health.

[7] Liao Y, Huang YM, Huang SH, Chen HC, Wei CW (2019). Exploring the switching intention of learners on social network-based learning platforms: a perspective of the push–pull–mooring model. Eurasia J Math Sci Tech Ed.

[8] Aikat J, Carsey TM, Fecho K, Jeffay K, Krishnamurthy A, Mucha PJ, Rajasekar A, Ahalt SC (2017). Scientific training in the era of Big Data: a New Pedagogy for Graduate Education. Big data.

[9] Wang YJ, Gao CL, Ye XD (2023). A data-driven precision teaching intervention mechanism to improve secondary school students’ learning effectiveness. Edu Inf Technol.

[10] Botvin M, Hershkovitz A (2023). Data-driven decision-making in emergency remote teaching. Edu Inf Technol.

[11] Fernandes JPS (2018). The importance of Medicinal Chemistry Knowledge in the clinical pharmacist’s education. Am J Pharm Educ.

[12] Klümper C, Neunzehn J, Wegmann U, Kruppke B, Joos U, Wiesmann HP (2016). Development and evaluation of an internet-based blended-learning module in biomedicine for university applicants–education as a challenge for the future. Head Face Med.

[13] Khan MO, Deimling MJ, Philip A (2011). Medicinal chemistry and the pharmacy curriculum. Am J Pharm Educ.

[14] Ramazan K, Devran AY, Muhammed ON (2024). An old approach to a novel problem: effect of combined balance therapy on virtual reality induced motion sickness: a randomized, placebo controlled, double-blinded study. BMC Med Educ.

[15] Singh J, Steele K, Singh L (2021). Combining the best of online and face-to-face learning: hybrid and blended Learning Approach for COVID-19, Post Vaccine, & Post-pandemic World. J Educ Technol Syst.

[16] Wu X (2021). Data-driven teaching–learning-based optimization (DTLBO) framework for expensive engineering problems. Struct Multidiscip Optim.

[17] Li J, Xiao C, Hou J, Zhao Y, Gong H, Zhang B, Yan M (2023). Clinical pharmacy undergraduate education in China: a comparative analysis based on ten universities’ training programs. BMC Med Educ.

[18] Li P, Zhang H, Tsai SB. A New Online and Offline Blended Teaching System of College English Based on Computer Internet Technology. Math Probl Eng 2021, 2021:3568386.

[19] Dumford AD, Miller AL (2018). Online learning in higher education: exploring advantages and disadvantages for engagement. J Comput High Educ.

