# Supplementary material for: Application of data-driven blended online-offline teaching in medicinal chemistry for pharmacy students: a randomized comparison
Source: BMC Med Educ. 2024 Jul 9;24:738. doi: 10.1186/s12909-024-05701-x (PMC11232250; doi:10.1186/s12909-024-05701-x)
Supplement: Supplementary file 1 — Supplementary Material 1 [file 12909_2024_5701_MOESM1_ESM.docx]

The questionnaire survey was designed independently and is detailed as follows:

1) The teaching approach you've adopted can increase learning interest.

A strongly agreed B agreed C neutral D disagreed E strongly disagreed

2)The teaching approach you've adopted can enhance learning target.

A strongly agreed B agreed C neutral D disagreed E strongly disagreed

3)The teaching approach you've adopted can enhance learning motivation.

A strongly agreed B agreed C neutral D disagreed E strongly disagreed

4) The teaching approach you've adopted can enhance one's self-learning ability.

A strongly agreed B agreed C neutral D disagreed E strongly disagreed

5) The teaching approach you've adopted can enhance one's mastery of basic knowledge.

A strongly agreed B agreed C neutral D disagreed E strongly disagreed

6) The teaching approach you've adopted can enhance teamwork skill.

A strongly agreed B agreed C neutral D disagreed E strongly disagreed

7) The teaching approach you've adopted can enhance one's problem-solving ability.

A strongly agreed B agreed C neutral D disagreed E strongly disagreed

8) The teaching approach you've adopted can enhance one's innovation ability.

A strongly agreed B agreed C neutral D disagreed E strongly disagreed

9) Your satisfaction with the teaching method.

A very satisfied B satisfied C neutral D dissatisfied
